# Clinical characteristics, drug resistance and death risk factors of *Burkholderia cepacia* infection in hematopoietic stem cell transplant patients

**DOI:** 10.1186/s12879-022-07754-z

**Published:** 2022-10-06

**Authors:** Yan Jia, Ya Liu, Yi Liu, Kaitai Yang, Yanfeng Liu

**Affiliations:** 1 Department of Hematology, Xiangya Hospital, Central South University, 87 Xiangya Road, Changsha, 410008 China; 2 National Clinical Research Center for Geriatric Disorders, Xiangya Hospital, Central South University, 87 Xiangya Road, Changsha, 410008 China; 3 Department of Gastroenterologyy, Xiangya Hospital, Central South University, 87 Xiangya Road, Changsha, China

**Keywords:** *Burkholderia cepacia*, Hematopoietic stem cell transplantation, Infection, Respiratory failure, Drug resistance

## Abstract

**Background:**

*Burkholderia cepacia* (BC) has been detected more and more in infected patients in recent years. However, as a high-risk population, the clinical characteristics and prognosis of BC infection in hematopoietic stem cell transplantation (HSCT) patients have not been reported. The purpose of this study is to obtain data that will help fill in the gaps in this field, provide evidence for reducing the mortality rate of BC infection in HSCT patients, and guide the use of antibiotics in the future.

**Methods:**

Electronic medical records of patients with BC infection who underwent HSCT in Xiangya Hospital of Central South University from September 1, 2015 to August 31, 2021 were collected. At the same time, 1:1 case–control matching was conducted according to gender, age and disease type. Comparisons between patients with/without BC infection and respiratory failure were made respectively, and the sensitivity of BC to five clinically commonly used antibiotics was also evaluated. Univariate and multivariate analyses were performed to identify independent risk factors for death.

**Results:**

The most common site of BC infection in HSCT patients was the lung (75%). Although BC infection rate (3.74%) and antibiotic resistance were not significant, it was closely associated with a higher risk of death (P = 0.022), which even further increased to 90.9% when combined with respiratory failure (P = 0.008). Procalcitonin > 10 µg/L (HR = 40.88, 95% CI 6.51–256.63, P = 0.000) and septic shock (HR = 4.08, 95% CI 1.02–16.33, P = 0.047) were two independent risk factors for death.

**Conclusion:**

HSCT patients with BC infection are in critical condition, and the management of respiratory infection should be especially strengthened to improve the prognosis of these patients.

## Introduction

Hematopoietic stem cell transplantation (HSCT) is an important treatment for hematological malignancies and bone marrow failure syndromes [1, 2], especially as the technology continues to mature and the problem of limited donor sources is solved (haploid HSCT), the coverage and success of transplantation therapy are getting higher and higher [3]. However, it should not be ignored that “infection” is still an important obstacle before the successful discharge of post-transplant patients due to the implementation of myeloablative pretreatment chemotherapy, the use of high-dose immunosuppressants, the destruction of patients’ own mucosal barrier, and the delay of hematopoietic reconstruction [4]. According to statistics, bacterial infection has always been the top complication after transplantation and can lead to a series of critical illnesses such as septic shock, multiple organ failure, and even death [5]. Not only that, but the prevalence and resistance of bacterial infection are constantly changing around the world. Different from the previous infection by Gram-positive cocci, Gram-negative bacilli have gradually become the main source of post-transplant infection in recent years. In particular, the increasing proportion of drug-resistant bacteria, which brings great difficulties to the treatment of patients [6].

*Burkholderia cepacia* (BC) is a Gram-negative non-fermentative bacterium commonly found in the natural environment, which mainly involving the respiratory system, urinary system, blood system and central nervous system. It is considered to be a high-risk opportunistic pathogen in patients with chronic granuloma, pulmonary cystic fibrosis, immunosuppression, etc., and can even cause outbreaks of nosocomial infection [7, 8]. With the increase of invasive diagnosis and treatment technology application, the extension of survival time of malignant tumor patients, and the upgrading of automatic microbial identification and analysis system, BC detection rate showing an upward trend [9], and clinical infection cases caused by this bacterium are also nothing new. BC has become another important non-fermentation infection bacterium after *Pseudomonas aeruginosa*, *Acinetobacter baumannii* and *Stenotrophomonas maltophilia*.

Up to now, studies on BC infection in transplant patients have mainly focused on lung transplantation for cystic fibrosis [10, 11], while investigations in other transplantation fields have rarely been involved or only a few cases have been reported [12]. This work is the first to systematically analyze the clinical characteristics, drug resistance and death risk factors of BC infection in HSCT patients, which aiming to obtain data that can help fill this gap, and provide basis for reducing the mortality rate of BC infection in this special population and guiding the use of antibiotics in the future.

## Methods

### Research design

Electronic medical record information of 32 HSCT patients with BC infection from September 1, 2015 to August 31, 2021 in the Department of Hematology, Xiangya Hospital, Central South University (Changsha, China) were collected, and a 1:1 retrospective case–control matching based on gender, age and disease type was performed. If more than one control patient met the inclusion criteria at the same time, the patient closest to the case was retained (controls with missing data were excluded). Clinical features included blood routine, liver and kidney function indexes at the time of infection, infection site, antibiotic treatment, hematopoietic engraftment time, septic shock, ICU admission, mechanical ventilation, survival, etc.

### Ethics

Due to the retrospective nature of this study, there was no interference in patients’ diagnosis and treatment, so the Ethics Committee of Xiangya Hospital approved this study to exempt patients from signing informed consent before data collection. This study complied with the Declaration of Helsinki and follows the principles of medical ethics. Authors kept all patients’ information strictly confidential.

### Definitions

The diagnosis and classification of diseases involved in this study were based on the “Chinese guidelines for the diagnosis and treatment of adult acute myeloid leukemia (not APL)”, “Guidelines for diagnosis and treatment of β-thalassemia major” and “The guidelines for diagnosis and treatment of chronic myelogenous leukemia in China” [13–16].

According to the criteria recommended by the U.S. Centers for Disease Control, BC infection is considered if pathogens are isolated from blood or other sterile specimens (urine, bronchoalveolar lavage fluid, pleural/abdominal fluid, deep wound secretions, etc.) with clear clinical manifestations of infection [17]. For bronchoalveolar lavage (BAL), the specific procedure was as follows: a fiberoptic bronchoscope (FB) was inserted through the artificial airway, and after exhausting respiratory secretions, FB was inserted into the opening of a severely diseased lobe/segment of bronchus, or the diseased lung segment shown on preoperative chest X-ray/CT. Lavage with 37 °C sterile physiological saline 100 mL in divided doses, 20 mL each time. The bronchoalveolar lavage fluid was collected with sterile tubes and sent for bacterial culture within 30 min. Septic shock is defined as persistent hypotension despite adequate volume resuscitation, vasoconstrictor drugs were still required to maintain mean arterial pressure (MAP) ≥ 65 mmHg, and serum lactate levels > 2 mmol/L [18]. Empiric anti-infective therapy refers to antibiotic treatment given within 48 h after the occurrence of suspected infection (drug susceptibility test results are not yet known) [19]. Crude mortality is defined as deaths from all causes within 100 days of infection.

### Microbial testing

Specimen collection was carried out by experienced nurses or doctors and sent for examination by special personnel within the specified time. The above process was strictly in accordance with the principles of aseptic operation. BC was identified by VITEK 2 compact automatic bacterial analyzer, drug susceptibility was determined by Kirby Bauer diffusion method (intermediate sensitivity was assessed as drug resistance).

### Statistical analysis

Statistical analysis was performed using IBM SPSS 24.0 software. Continuous variables were expressed as mean ± standard deviation or quartile, categorical variables were described by absolute numbers and percentages. Comparison between groups was performed by Student’s t-test, Mann–Whitney U test, Chi-square test, or Fisher’s exact test. Univariate analysis was used to assess the association between clinical/epidemiological variables and crude mortality, where variables with P < 0.05 were entered into multivariate Cox regression analysis to identify independent risk factors. Meanwhile, hazard ratios (HR) and 95% confidence interval (CI) were calculated. Kaplan–Meier method was used to draw the survival curve, and log-rank test was used to compare the survival analysis of risk factors. P < 0.05 was considered statistically significant.

## Results

### Clinical characteristics of HSCT patients with BC infection

BC infection occurred in 32 of 855 patients (3.74%) who underwent HSCT for hematological diseases during the 6-year study period, including 25 (78.1%) men and 7 (21.9%) women, with a mean age of 19.62 years. The disease types involved included acute myeloid leukaemia (25%), thalassemia (56.2%) and chronic myelogenous leukaemia (18.8%). Lung was the most common site of infection (n = 24), and the incidence of acute graft-versus-host disease (aGVHD) after transplantation was 78.1%. Compared with matched control, patients with BC infection not only had significantly higher rates of thrombocytopenia (P = 0.043), renal impairment (P = 0.024), multi-site/pathogen infection (P = 0.018), anti-CMV immunoglobulin use (P < 0.001), ICU admission (P < 0.001), and septic shock (P = 0.012), but also a worse prognosis (mortality 56.2% vs. 25.0%, P = 0.022) (Table 1).

**Table 1:** Clinical characteristics and comparison between with and without BC infections in HSCT patients

Characteristic	Total	With BC (n = 32)	Without BC (n = 32)	P-value
Age, years (mean ± SD)	19.16 ± 15.31	19.62 ± 16.33	18.69 ± 14.47	0.809
Gender, n (%)				0.274
Male, n (%)	45 (70.3)	25 (78.1)	20 (62.5)	
Female, n (%)	19 (29.7)	7 (21.9)	12 (37.5)	
Primary disease, n (%)				1.000
Acute myelogenous leukemia	16 (25)	8 (25)	8 (25)	
Thalassemia	36 (56.2)	18 (56.2)	18 (56.2)	
Chronic myelogenous leukemia	12 (18.8)	6 (18.8)	6 (18.8)	
Infection site, n (%)				0.781
Pulmonary infection	46 (71.9)	24 (75)	22 (68.8)	
Bloodstream infection	18 (28.1)	8 (25)	10 (31.2)	
aGVHD, n (%)	55 (85.9)	25 (78.1)	30 (93.8)	0.148
Catheter, n (%)	4 (6.2)	2 (6.2)	2 (6.2)	1.000
Indicators of initial stage of infection				
Neutrophil count < 0.5 × 10^9^/L, n (%)	14 (21.9)	4 (12.5)	10 (31.2)	0.131
Platelet count < 20 × 10^9^/L, n (%)	16 (25)	4 (12.5)	12 (37.5)	0.043*
Creatinine > 177 µmol/L, n (%)	6 (9.4)	6 (18.8)	0 (0)	0.024*
Total bilirubin > 34.2 µmol/L, n (%)	14 (21.9)	6 (18.8)	8 (25)	0.762
Hematopoietic engraftment, days (mean ± SD)	18.5 ± 8.53	19.97 ± 10.3	17.03 ± 6.12	0.17
Multiple site/pathogen infection after transplantation, n (%)	22 (34.4)	16 (50)	6 (18.8)	0.018*
Use of anti-CMV immunoglobulin after transplantation, n (%)	24 (37.5)	20 (62.5)	4 (12.5)	< 0.001*
Admission to ICU after transplantation, n (%)	26 (40.6)	20 (62.5)	6 (18.8)	< 0.001*
Mechanical ventilation, n (%)	17 (26.6)	11 (34.4)	6 (18.8)	0.258
Septic shock, n (%)	18 (28.1)	14 (43.8)	4 (12.5)	0.012*
Mortality, n (%)	26 (40.6)	18 (56.2)	8 (25.0)	0.022*

*SD* standard deviation, *aGVHD* acute graft-versus-host disease, *CMV* cytomegalovirus, *ICU* intensive care unit

*P values are statistically significant between with and without BC infection group

### Comparison of BC infected patients with and without respiratory failure (RF)

As can be seen from Table 2, among BC infected patients with or without respiratory failure, gender, age, infection site, use of broad-spectrum antibiotics for more than 5 days within 1 month before transplantation, cytomegalovirus (CMV) infection before transplantation, HLA matching degree, hematopoietic engraftment time after transplantation, whether mesenchymal stem cells were injected, whether GVHD occurred, and some biochemical indicators in the initial stage of infection (neutrophil and platelet counts, procalcitonin (PCT), albumin, total bilirubin, creatinine) had no statistical difference between groups (P > 0.05). However, patients who developed respiratory failure were significantly more severe and had a worse prognosis than those who did not (mortality 90.9% vs. 38.1%, P = 0.008).

**Table 2:** Comparison of 32 BC infected HSCT patients with and without respiratory failure

Characteristic	Without RF (n = 21)	With RF (n = 11)	P-value
Age, years, meidan (IQR)	14 (7, 47)	14 (7, 30)	0.968
Gender, n (%)			0.374
Male	15 (71.4)	10 (90.9)	
Female	6 (28.6)	1 (9.1)	
Infection site, n (%)			0.681
Pulmonary infection	15 (71.4)	9 (81.8)	
Bloodstream infection	6 (28.6)	2 (18.2)	
CMV infection before transplantation, n (%)	7 (33.3)	5 (45.5)	0.703
Use of broad-spectrum antibiotics > 5 days 1 month prior to infection, n (%)	19 (90.5)	9 (81.8)	0.593
HLA identical match, n (%)	15 (71.4)	5 (45.5)	0.250
Mesenchymal stem cell infusion, n (%)	4 (19)	0 (0)	0.272
Hematopoietic reconstruction time > 15 days, n (%)	8 (38.1)	6 (54.5)	0.465
aGVHD, n (%)	15 (71.4)	10 (90.9)	0.374
Indicators of initial stage of infection			
Neutrophil count, 10^9^/L, median (IQR)	1.6 (0.90, 2.00)	3.4 (1.10, 8.80)	0.196
Platelet count, 10^9^/L, median (IQR)	33 (23.00, 41.00)	46 (33.00, 151.00)	0.256
PCT, µg/L, median (IQR)	0.67 (0.20, 2.16)	34.29 (0.05, 55.21)	0.349
Albumin, g/L, median (IQR)	35.6 (31.30, 37.10)	29 (29.00, 36.90)	0.735
Total bilirubin, µmol/L, median (IQR)	13.8 (9.20, 36.00)	9.5 (7.30, 11.90)	0.064
Creatinine, µmol/L, median (IQR)	47 (43.80, 98.00)	84 (41.8, 98.00)	0.511
Irrational empiric anti-infective therapy, n (%)	5 (23.8)	5 (45.5)	0.252
Admission to ICU after transplantation, n (%)	9 (42.9)	11 (100)	0.002*
Renal replacement therapy, n (%)	3 (14.3)	5 (45.5)	0.088
Septic shock, n (%)	5 (23.8)	9 (81.8)	0.003*
Mortality, n (%)	8 (38.1)	10 (90.9)	0.008*

*HLA* human leukocyte antigen, *IQR* interquartile range, *PCT* procalcitonin

*P values are statistically significant between with and without respiratory failure group

### Antibiotic susceptibility analysis of BC

Table 3 shows the susceptibility of BC infected HSCT patients to five clinically commonly used antibiotics. In general, BC had low drug resistance rates to cefoperazone–sulbactam, ceftazidime, meropenem, cotrimoxazole and minocycline, but there were slight differences in different infection sites. For example, cefoperazone–sulbactam and minocycline were significantly more effective in treating pulmonary infection than bloodstream infection, but ceftazidime was more sensitive to bloodstream infection than pulmonary infection.

**Table 3:** Resistance of BC strains in different infection sites to 5 antibiotics commonly used in clinic

Antimicrobial	Bloodstream infection (n = 10)	Pulmonary infection (n = 22)	Total (n = 32)
Cefoperazone–sulbactam, n (%)	7 (70)	5 (22.7)	13 (40.6)
Ceftazidime, n (%)	1 (10)	6 (27.3)	7 (21.9)
Meropenem, n (%)	2 (20)	2 (9)	4 (12.5)
Cotrimoxazole, n (%)	0 (0)	4 (18.2)	4 (12.5)
Minocycline, n (%)	6 (60)	6 (27.3)	12 (37.5)

### Risk factors for death from BC infection

**Fig. 1:**
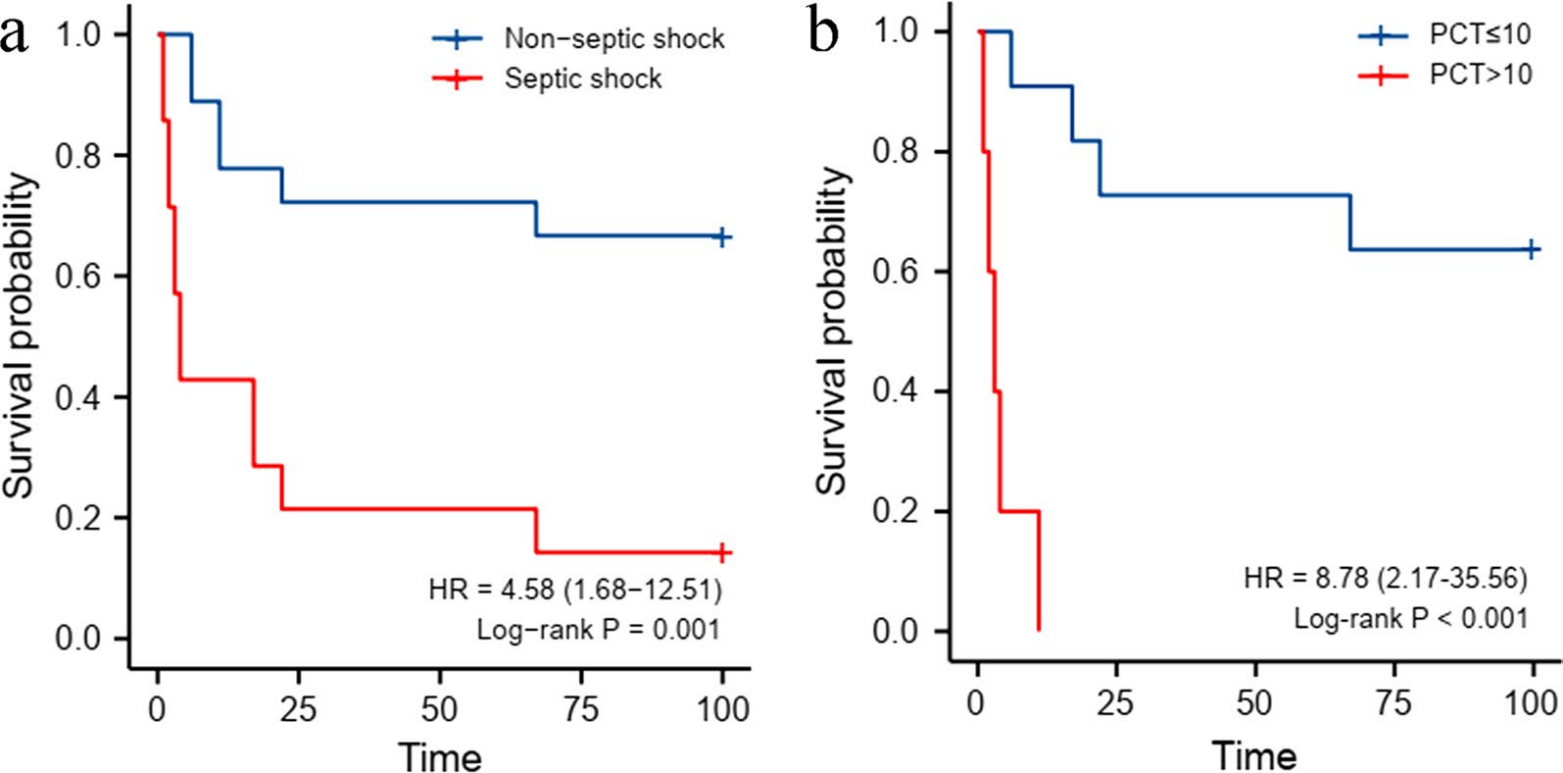
Survival estimated by Kaplan–Meier curve: **A** septic shock vs. non-septic shock (14.3% vs. 66.7%, P = 0.001); **B** PCT > 10 µg/L vs. PCT ≤ 10 µg/L (0% vs. 63.6%, P < 0.001)

Comparison between the death group and the survival group is shown in Table 4. In univariate analysis, risk factors associated with death included aGVHD (P = 0.027), PCT > 10 ng/L (P = 0.001), irrational empiric anti-infective therapy (P = 0.019), mechanical ventilation (P = 0.008), and septic shock (P = 0.005). In multivariate analysis, PCT > 10 µg/L (HR = 40.88, 95% CI 6.51–256.63, P = 0.000) and septic shock (HR = 4.08, 95% CI 1.02–16.33, P = 0.047) were identified as two independent risk factors for death. Figure 1 shows the survival of patients with independent risk factors: the survival rate of patients with septic shock was significantly lower than that of patients without septic shock (14.3% vs. 66.7%, P = 0.001); and compared with PCT > 10 ng/L group, the survival rate of patients with PCT ≤ 10 µg/L was higher ( 63.6% vs. 0%, P < 0.001).

**Table 4:** Univariate and multivariate analysis of risk factors for mortality in HSCT patients with BC infection

Variable	Survival (n = 14)	Mortality (n = 18)	Univariate analysis	Multivariate analysis		
			P-value	HR (95% CI)		P-value
Gender, n (%)			0.195			
Male, n (%)	9 (64.3)	16 (88.9)		–		–
Female, n (%)	5 (35.7)	2 (11.1)				
Age < 14 or ≥ 50 years, n (%)	10 (71.4)	7 (38.9)	0.087	–		–
Hematopoietic reconstruction time > 15 days, n (%)	6 (42.9)	8 (44.4)	1.000	–		–
aGVHD, n (%)	8 (57.1)	17 (94.4)	0.027*	1.68 (0.18–15.24)		0.646
Total bilirubin > 34.2 µmol/L, n (%)	2 (14.3)	4 (22.2)	0.672	–		–
PCT > 10 µg/L, n (%)	0 (0)	10 (55.6)	0.001*	40.88 (6.51-256.63)		0.000*
Irrational empiric anti-infective therapy, n (%)	1 (7.1)	9 (50)	0.019*	2.13 (0.62–7.34)		0.231
Mechanical ventilation, n (%)	1 (7.1)	10 (55.6)	0.008*	2.75 (0.75–10.09)		0.127
Septic shock, n (%)	2 (14.3)	12 (66.7)	0.005*	4.08 (1.02–16.33)		0.047*
Admission to ICU, n (%)	6 (42.9)	14 (77.8)	0.068	–		–

*P values are statistically significant

## Discussion

BC is widely found in water, soil and plants in nature. It can survive for a long time and spread through contact or respiratory tract. The main pathogenic factor is adhesin, which is an important pathogen causing nosocomial infection [20]. If not controlled promptly, BC infection can not only cause severe pneumonia (sometimes even develop into “cepacia syndrome” leading to respiratory failure), but also result in systemic infections such as bacteremia and sepsis [21, 22]. In the past, for the infection of patients after HSCT, people might pay more attention to *Klebsiella pneumoniae*, *Escherichia coli*, *Acinetobacter baumannii*, *Pseudomonas aeruginosa*, etc. [23]. Here, our survey found that although the detection rate of BC was indeed lower than that of the above-mentioned bacteria—only less than 5%, which is basically consistent with the data reported by Lu et al. [24]. However, the resulting risk of death is greatly increased, thus placing a heavy burden on the life and property safety of patients. Age, ICU stay more than 2 weeks, APACHE II score, and the presence of efflux pump gene were reported to be independent risk factors for developing BC bloodstream infection [25]. A previous study showed that due to the obviously increased mortality in patients with BC infection, in the early days, some medical institutions even held reservations about whether patients with pulmonary cystic fibrosis complicated by BC infection were suitable for lung transplantation [26, 27]. To our knowledge, this is the first retrospective case–control study to specifically investigate the impact of BC on HSCT patients, and proposed that BC infection is associated with septic shock, admission to ICU for intensive care, and renal impairment.

Our study shows that the lung remains the most common site of BC infection. On this basis, pulmonary rejection after HSCT may further increase the likelihood of lung function deterioration and respiratory failure, although we have not found statistical differences in the occurrence of aGVHD between the BC infected group and the matched non-BC infected group. However, it still reminds us of the importance of strengthening the management of respiratory infection during transplantation. In addition, we also found that CMV infection before transplantation may be a related factor to induce BC infection. CMV has been reported to have significant bone marrow suppression [28]. The replication and reactivation of it can initiate systemic inflammatory response, affect hematopoiesis and immune reconstitution, which may create opportunities for BC invasion [29]. The higher frequency of use of anti-CMV immunoglobulin in the BC group after transplantation also supports this view.

Unexpectedly, despite the high mortality rate, BC infection did not show obvious resistance to a variety of commonly used antibiotics, which is similar to the results of multiple previous studies [30, 31]. We speculate that this may be related to the improper selection of antibiotics during empiric anti-infective therapy. For the post-transplant population, considering factors such as immunosuppression, catheter implantation, high venous nutrition and delayed hematopoietic reconstruction, clinicians often choose to “strike hard” at the beginning of infection. In contrast, ceftazidime, levofloxacin, cotrimoxazole and other seemingly “weak” or relatively high resistance antibiotics were excluded. Another possibility is that HSCT patients are usually accompanied by multi-pathogen infection, so even if some patients choose antibiotics sensitive to BC, such as meropenem, cefoperazone sulbactam, etc., they still have poor anti-infection effect and eventually die under the combined action of other drug-resistant bacteria.

Septic shock and PCT > 10 µg/L are independent risk factors associated with death that we obtained in this study. Septic shock is a serious systemic disease in which microorganisms and their metabolites invade the blood circulation, activate the host immune system, produce cytokines and endogenous mediators, and then act on various organs/systems and affect perfusion, eventually leading to cell ischemia and hypoxia, metabolic disorder and multiple organ dysfunction (MODS) [32]. The clinical treatment of sepsis and septic shock is a huge challenge that threatens global health. The World Health Organization (WHO) advocates that governments should make it a “diseases of global medical priority concern”. According to the recommendations of SSC Guide for shork (2021 version), screening and early therapy, hemodynamic management, mechanical ventilation, long-term care and support are treatment priorities [33]. Since septic shock is a complex and changing process of interaction between microorganisms and the body, it is highly heterogeneous from pathogenic bacteria to early systemic inflammatory response syndrome (SIRS) and compensatory anti-inflammatory response syndrome (CARS). Therefore, individualized intervention at different stages is needed [34]. Patients with BC infection after HSCT, as a special population with severely suppressed immune function, should pay more attention to the clinical management of septic shock. The first is the identification of risk factors, such as older age, malnutrition, hyperthermia or hypothermia, prolonged hospital stay, unstable vital signs (heart rate > 120 beats/min, systolic blood pressure < 110 mmHg or 60–70% below baseline), central venous catheter, long-term use of antibiotics/hormones/chemotherapy drugs, viral infections, etc. The second is the rapid assessment of the shock, that is, according to clinical manifestations to determine the patient’s stage (shock compensation or decompensation). Finally, use antibiotics empirically as soon as possible, and actively identify the type of pathogenic bacteria (blood culture, sputum culture, NGS and other methods). At the same time, based on the analysis of pathophysiology of patients, reduce the inflammatory response and improve organ function, so as to prevent the development of septic shock to MODS.

PCT has been identified as an important biomarker for diagnosing infection (especially excellent in the early diagnosis of infections caused by Gram-negative strains), and PCT > 10 µg/L is more indicative of the severity of bacterial infection or the insufficiency of antibacterial treatment, because a large number of studies have confirmed that the significantly increased PCT is positively correlated with poor prognosis of patients [35–37]. For example, as early as 1993 Assicot et al. reported that PCT levels were directly related to the extent and severity of microbial invasion [38]. Later, Dan et al. also found that the PCT of infected patients in the death group was significantly higher than that in the control group [39]. In addition, Muller and Hina et al. also pointed out that elevated levels of PCT were associated with increased infection severity in community-acquired pneumonia [40, 41].

Although our conclusions are preliminary, and limited by retrospective study design and factors such as sample size, completeness of medical record information, selection bias, etc., our study is the first to provide clinicians with useful information on the patterns, characteristics, mortality risk factors and antibiotic susceptibility of BC infection in HSCT patients. Continuing to expand the sample size and combine multi-centers in different regions to conduct prospective studies will be the focus of our next research.

## Conclusion

The most common site of *Burkholderia cepacia* infection in HSCT patients is the lung. Despite the low incidence, HSCT patients with BC infection have a significantly poorer prognosis. Septic shock and PCT > 10 ng/L are two independent risk factors for death. The sensitivity of BC to common clinical antibiotics is generally good, but there are slight differences in different infection sites. It is recommended to choose cefoperazone-sulbactam and minocycline for pulmonary infection. Although our findings are preliminary, and there are some limitations to our study (such as sample size, completeness of medical record information, selection bias, etc.). However, it is undeniable that our research fills a gap in the field of clinical characteristics of BC infection in HSCT patients and provides valuable reference data for improving the overall efficacy of this special population in the future.

## Data Availability

All data generated or analysed during this study are available from the corresponding author on reasonable request.

## Abbreviations

BC: *Burkholderia cepacia*
HSCT: Hematopoietic stem cell transplantation
HR: Hazard ratios
CI: Confidence interval
aGVHD: Acute graft-versus-host disease
CMV: Cytomegalovirus
PCT: Procalcitonin
RF: Respiratory failure
ICU: Intensive care unit
